# Use of prokinetic agents in hospitalised adult patients: Protocol for a scoping review

**DOI:** 10.1111/aas.14099

**Published:** 2022-06-22

**Authors:** Vera Crone, Morten Hylander Møller, Anders Perner, Waleed Alhazzani, Mette Krag

**Affiliations:** ^1^ Department of Intensive Care Holbæk Hospital Holbæk Denmark; ^2^ Department of Intensive Care, Rigshospitalet University of Copenhagen Copenhagen Denmark; ^3^ Collaboration for Research in Intensive Care (CRIC) Copenhagen Denmark; ^4^ Department of Medicine McMaster University Hamilton Ontario Canada; ^5^ Department of Clinical Epidemiology and Biostatistics McMaster University Hamilton Ontario Canada; ^6^ Department of Clinical Medicine University of Copenhagen Copenhagen Denmark

## Abstract

**Background:**

Gastrointestinal motility is an important contributor to the effective uptake of water and nutrition. However, it is often impaired in acutely ill hospitalised patients. Amongst other indications, prokinetic agents are used to improve GI motility, but the body of evidence is not well described. Accordingly, we aim to systematically describe and explore the body of evidence on the use of prokinetic agents in hospitalised adults.

**Methods:**

In accordance with the Preferred Reporting Items for Systematic Reviews and Meta‐Analyses extension for Scoping Reviews statement, we plan to conduct a scoping review of studies assessing the use of prokinetic agents, for any indication, in hospitalised adults. We plan to assess study design, population, agents, indications and outcomes across included studies. When applicable, we plan to assess the certainty of evidence using the Grading of Recommendations Assessment, Development and Evaluation (GRADE) approach.

**Results:**

We plan to provide descriptive analyses of the included studies accompanied by tabulated results and characterise knowledge gaps.

**Conclusion:**

The outlined scoping review will provide a summary of the body of evidence on the use, indications, effects and side effects of prokinetic agents in hospitalised adults.

## 1 BACKGROUND

Sufficient uptake of water and nutrition is essential for hospitalised patients to prevent malnutrition. Malnutrition is associated with increased morbidity and mortality.^1^ However, delivery of sufficient nutrition depends largely on gastrointestinal (GI) motility, which is often impaired in hospitalised patients due to adverse drug effects, hyperglycaemia, immobility, impaired renal function, mechanical ventilation, or as part of the underlying disease.^2^, ^3^ Delayed gastric emptying can result in retention of gastric content, vomiting, diarrhoea, pneumonia and insufficient absorption of nutrients.^2^ Clinicians use prokinetic agents to improve GI motility.^4^ Prokinetic agents are also used for other indications in hospitalised patients, including nausea and vomiting, pseudo‐obstruction, functional dyspepsia, gastroparesis and to improve visualisation during gastroscopy.^4^, ^5^, ^6^, ^7^

Several prokinetic agents with different mechanisms of action exist, including dopamine receptor antagonists, motilin receptor agonists, serotonin (5‐hydroxytryptamine type 4) receptor agonists, cholinesterase inhibitors and ghrelin agonists.^8^, ^9^

We aim to systematically outline and explore the body of evidence on the use of prokinetic agents for any indication in hospitalised adults. We hypothesise that prokinetic agents are used in a wide range of conditions and that the quantity and quality of evidence are low.

## 2 METHODS

This protocol has been prepared according to the Preferred Reporting Items for Systematic Review and Meta‐Analysis Protocol (PRISMA‐P) statement.^10^

The outlined review will be prepared and reported according to the Preferred Reporting Items for Systematic Reviews and Meta‐Analyses extension for Scoping Reviews (PRISMA‐ScR).^11^

### 2.1 Research questions

- Which hospitalised adult patient populations receive prokinetic agents?
- What prokinetic agents are used?
- What are the indications for the use of prokinetic agents?
- Which desirable and undesirable outcomes have been assessed?

### 2.2 Types of studies

We will include all studies regardless of publication source, status, language and study design.

### 2.3 Types of participants

We will include studies on hospitalised adult patients receiving prokinetic agents for any indication. Studies in animals and children, as well as studies in healthy subjects, will be excluded.

### 2.4 Intervention and comparator

We aim to include studies reporting on any type of prokinetic agents given for any indication including studies that compared prokinetic agents to each other or no treatment/placebo.

### 2.5 Types of outcome measures

All reported outcome measures will be described.

### 2.6 Electronic searches

We will systematically search the following databases: Medline, EMBASE, Cochrane Library and Epistemonikos. Additionally, we will search for ongoing trials on Clinicaltrials.gov. A tentative search strategy for Medline is available in the supplement. If we detect additional relevant keywords during the search process, we will include these in the search strategy and document the changes. Before submitting the final review draft, we will conduct an updated search and include any relevant records. We will manually search the reference list for relevant studies and other systematic reviews on the subject. Unpublished trials will be sought identified and authors will be contacted for additional data, if relevant.

### 2.7 Data collection and analysis

#### 2.7.1 Selection of studies

At least two review authors will independently screen the title and abstract of all studies identified in the search. All potentially relevant records will be assessed in full text for eligibility. Discrepancies will be resolved through consensus or discussion with a third reviewer. We will present a PRISMA flowchart of the study selection process.^12^

#### 2.7.2 Data extraction and management

Two review authors will extract information from the included studies using a predesigned data extraction form. The extracted information will include trial characteristics (type of study, year of publication and country) characteristics of participants, type of intervention and type of comparator (for comparative studies) and outcomes.

#### 2.7.3 Strategy for data synthesis

We will present the results descriptively accompanied by tabulated results. Studies will be grouped according to study design, to explore any heterogeneity due to differences in design. We will not provide a detailed assessment or critical appraisal of the individual studies.

#### 2.7.4 Quality of evidence

We will use the Grading of Recommendations Assessment, Development and Evaluation (GRADE) approach to assess the certainty of evidence.^13^ The domains assessed will be a risk of bias, inconsistency (heterogeneity), indirectness (including other patient populations or use of surrogate outcomes), imprecision (wide confidence interval around the effect estimate or a low number of included patients) and publication bias. The overall certainty of evidence will be judged as high, moderate, low or very low.

## 3 DISCUSSION

The outlined scoping review will provide an overview of the body of evidence on the use of prokinetic agents in hospitalised adult patients and highlight gaps in knowledge.

Prokinetic agents are used in hospitalised patients, but the extent and indications are not well described.

The strengths of the planned scoping review include the predefined protocol, the systematic search, compliance with PRISMA statements^10^, ^12^, ^14^ and assessment of the certainty of evidence according to GRADE.^13^ The planned review has some limitations. We will not assess the risk of bias in individual studies. Furthermore, we expect some clinical heterogeneity amongst the included studies regarding population, setting and intervention.

## 4 CONCLUSION

The proposed scoping review will provide an overview of the current evidence on the use of prokinetic agents in hospitalised adult patients, and research priorities will be identified.

## CONFLICT OF INTEREST

None.

## Supporting information


Data S1
