# Supplementary material for: Use of prokinetic agents in hospitalised adult patients: Protocol for a scoping review
Source: Acta Anaesthesiol Scand. 2022 Jun 22;66(8):1024–6. doi: 10.1111/aas.14099 (PMC9545779; doi:10.1111/aas.14099)
Supplement: Supplementary file 1 — Data S1 [file AAS-66-1024-s001.docx]

**Search Strategy Medline**

| 1. |  | Critical Care/ |
| --- | --- | --- |
| 2. |  | Critical Illness/ or Intensive Care Units/ |
| 3. |  | Acute Disease/ |
| 4. |  | Coronary Care Units/ |
| 5. |  | Burn Units/ |
| 6. |  | Respiratory Care Units/ |
| 7. |  | 1 or 2 or 3 or 4 or 5 or 6 |
| 8. |  | Sepsis/ |
| 9. |  | Bacteremia/ |
| 10. |  | Hemorrhagic Septicemia/ |
| 11. |  | Shock, Septic/ |
| 12. |  | Endotoxins/ |
| 13. |  | Multiple Organ Failure/ or Systemic Inflammatory Response Syndrome/ |
| 14. |  | Toxemia/ or Bacterial Toxins/ |
| 15. |  | 8 or 9 or 10 or 11 or 12 or 13 or 14 |
| 16. |  | 7 or 15 |
| 17. |  | (critical adj3 care).ab,kf,ti. |
| 18. |  | surgical intensive care.ab,kf,ti. |
| 19. |  | (intensive adj3 care).ab,kf,ti. |
| 20. |  | "critical illness*".ab,kf,ti. |
| 21. |  | (critical* adj3 ill).ab,kf,ti. |
| 22. |  | (acute adj3 disease).ab,kf,ti. |
| 23. |  | "catastrophic illness*".ab,kf,ti. |
| 24. |  | (intensive adj3 care unit*).ab,kf,ti. |
| 25. |  | (Coronary adj3 care unit*).ab,kf,ti. |
| 26. |  | (burn adj3 care unit*).ab,kf,ti. |
| 27. |  | (respiratory adj3 care unit*).ab,kf,ti. |
| 28. |  | 17 or 18 or 19 or 20 or 21 or 22 or 23 or 24 or 25 or 26 or 27 |
| 29. |  | sepsis.ab,kf,ti. |
| 30. |  | bacteremia.ab,kf,ti. |
| 31. |  | "bloodstream infection*".ab,kf,ti. |
| 32. |  | "pyemia*".ab,kf,ti. |
| 33. |  | "pyohemia*".ab,kf,ti. |
| 34. |  | "septicemia*".ab,kf,ti. |
| 35. |  | "blood poisoning*".ab,kf,ti. |
| 36. |  | (servere adj3 sepsis).ab,kf,ti. |
| 37. |  | (septic adj3 shock).ab,kf,ti. |
| 38. |  | "toxic shock syndrome*".ab,kf,ti. |
| 39. |  | (endotoxin adj3 shock*).ab,kf,ti. |
| 40. |  | "systemic inflammatory response syndrome*".ab,kf,ti. |
| 41. |  | (sepsis adj3 syndrome*).ab,kf,ti. |
| 42. |  | "toxeamia*".ab,kf,ti. |
| 43. |  | "toxemia*".ab,kf,ti. |
| 44. |  | (septic adj3 fever).ab,kf,ti. |
| 45. |  | (septic adj3 infection*).ab,kf,ti. |
| 46. |  | "multiple organ dysfunction syndrome*".ab,kf,ti. |
| 47. |  | "multiple organ failure*".ab,kf,ti. |
| 48. |  | 29 or 30 or 31 or 32 or 33 or 34 or 35 or 36 or 37 or 38 or 39 or 40 or 41 or 42 or 43 or 44 or 45 or 46 or 47 |
| 49. |  | 28 or 48 |
| 50. |  | 16 or 49 |
| 51. |  | (prokinetic adj3 agent*).ab,kf,ti. |
| 52. |  | "prokinetic*".ab,kf,ti. |
| 53. |  | (gastrointestinal adj3 agent*).ab,kf,ti. |
| 54. |  | (gastrointestinal adj3 drug*).ab,kf,ti. |
| 55. |  | "metoclopramid*".ab,kf,ti. |
| 56. |  | "maxolon*".ab,kf,ti. |
| 57. |  | "rimetin*".ab,kf,ti. |
| 58. |  | primperan.ab,kf,ti. |
| 59. |  | reglan.ab,kf,ti. |
| 60. |  | cerucal.ab,kf,ti. |
| 61. |  | (dopamin* adj3 antagonist*).ab,kf,ti. |
| 62. |  | "domperidon*".ab,kf,ti. |
| 63. |  | "domperidone monohydrochlorid*".ab,kf,ti. |
| 64. |  | "metoclopramide dihydrochlorid*".ab,kf,ti. |
| 65. |  | "metoclopramide hydrochlorid*".ab,kf,ti. |
| 66. |  | "metoclopramide monohydrochlorid*".ab,kf,ti. |
| 67. |  | "Erythromycin*".ab,kf,ti. |
| 68. |  | erythromycin A.ab,kf,ti. |
| 69. |  | "erythromycin phosphat*".ab,kf,ti. |
| 70. |  | "erythromycin lactat*".ab,kf,ti. |
| 71. |  | erythromycin C.ab,kf,ti. |
| 72. |  | (motility adj3 agent*).ab,kf,ti. |
| 73. |  | (motilin adj3 agonist*).ab,kf,ti. |
| 74. |  | "antiemetic agent*".ab,kf,ti. |
| 75. |  | "antiemetic drug*".ab,kf,ti. |
| 76. |  | relamorelin.ab,kf,ti. |
| 77. |  | Ulimorelin.ab,kf,ti. |
| 78. |  | (ghrelin adj3 agonist*).ab,kf,ti. |
| 79. |  | "cisaprid*".ab,kf,ti. |
| 80. |  | "mosaprid*".ab,kf,ti. |
| 81. |  | "itoprid*".ab,kf,ti. |
| 82. |  | (Serotonin 5-HT4 adj3 agonist*).ab,kf,ti. |
| 83. |  | TAK-954.ab,kf,ti. |
| 84. |  | felcisetrag.ab,kf,ti. |
| 85. |  | 51 or 52 or 53 or 54 or 55 or 56 or 57 or 58 or 59 or 60 or 61 or 62 or 63 or 64 or 65 or 66 or 67 or 68 or 69 or 70 or 71  or 72 or 73 or 74 or 75 or 76 or 77 or 78 or 79 or 80 or 81 or 82 or 83 or 84 |
| 86. |  | Gastrointestinal Agents/ or Domperidone/ or Gastrointestinal Motility/ |
| 87. |  | Gastric Emptying/ |
| 88. |  | Metoclopramide/ |
| 89. |  | Dopamine D2 Receptor Antagonists/ |
| 90. |  | Dopamine Antagonists/ |
| 91. |  | Erythromycin Estolate/ or Erythromycin/ or Erythromycin Ethylsuccinate/ |
| 92. |  | Receptors, Gastrointestinal Hormone/ or Motilin/ |
| 93. |  | Antiemetics/ |
| 94. |  | Receptors, Ghrelin/ |
| 95. |  | Ghrelin/ |
| 96. |  | Cisapride/ |
| 97. |  | Serotonin 5-HT4 Receptor Agonists/ |
| 98. |  | Gastrointestinal Transit/ |
| 99. |  | 86 or 87 or 88 or 89 or 90 or 91 or 92 or 93 or 94 or 95 or 96 or 97 or 98 |
| 100. |  | 85 or 99 |
| 101. |  | Hospitalization/ |
| 102. |  | Adolescent, Hospitalized/ |
| 103. |  | Patient Admission/ |
| 104. |  | Inpatients/ |
| 105. |  | 101 or 102 or 103 or 104 |
| 106. |  | "hospitaliz* adolescent*".ab,kf,ti. |
| 107. |  | "hospitaliz*".ab,kf,ti. |
| 108. |  | warded.ab,kf,ti. |
| 109. |  | "admitted to hospital*".ab,kf,ti. |
| 110. |  | admitted.ab,kf,ti. |
| 111. |  | "admission*".ab,kf,ti. |
| 112. |  | "inpatient*".ab,kf,ti. |
| 113. |  | 106 or 107 or 108 or 109 or 110 or 111 or 112 |
| 114. |  | 105 or 113 |
| 115. |  | 50 or 114 |
| 116. |  | 100 and 115 |
| 117. |  | Neostigmine/ |
| 118. |  | neostigmin.ab,kf,ti. |
| 119. |  | 51 or 52 or 53 or 54 or 55 or 56 or 57 or 58 or 59 or 60 or 61 or 62 or 63 or 64 or 65 or 66 or 67 or 68 or 69 or 70 or 71 or 72  or 73 or 74 or 75 or 76 or 77 or 78 or 79 or 80 or 81 or 82 or 83 or 84 or 86 or 87 or 88 or 89 or 90 or 91 or 92 or 93 or 94  or 95 or 96 or 97 or 98 or 117 or 118 |
| 120. |  | 115 and 119 |
